# A Comparison Study on the Magneto-Responsive Properties and Swelling Behaviors of a Polyacrylamide-Based Hydrogel Incorporating with Magnetic Particles

**DOI:** 10.3390/ijms222212342

**Published:** 2021-11-15

**Authors:** Chanchan Xu, Bin Li, Xiaojie Wang

**Affiliations:** 1 Institute of Intelligent Machines, Hefei Institutes of Physical Science, Chinese Academy of Sciences, Hefei 230000, China; xuchan@mail.ustc.edu.cn (C.X.); binli@iamt.ac.cn (B.L.); 2 Department of Precision Machinery and Instrumentation, University of Science and Technology of China, Hefei 230000, China

**Keywords:** magnetic hydrogels, magneto-rheology, magnetite, carbonyl iron, water-swelling

## Abstract

This work investigates the mechanical properties, microstructures, and water-swelling behavior of a novel hydrogel filled with magnetic particles. The nanoparticles of magnetite (Fe_3_O_4_) and the micro-particles of carbonyl iron (CI) were selected and filled into a polyacrylamide (PAAM) hydrogel matrix to create two types of magnetic hydrogels. The isotropy and anisotropy of magnetic hydrogels are also presented in this study. The isotropic samples were cured without applying a magnetic field (MF), and the anisotropic samples were cured by applying an MF in the direction perpendicular to the thickness of the samples. The effects of the size, content, and inner structures of magnetic particles on the magneto-responsive and swelling properties of magnetic hydrogels were investigated. It was found that the magnetorheological (MR) effect of anisotropic samples was apparently higher than that of isotropic samples, and the hydrogels with CI exhibited a noticeable MR effect than those with Fe_3_O_4_. The storage modulus can be enhanced by increasing the filler content and size, forming an anisotropic structure, and applying an external MF. In addition, the magnetic hydrogels also have a swelling ability that can be tuned by varying the content and size of the particle fillers.

## 1. Introduction

Hydrogels are formed by hydrophilic polymer chains with three-dimensional (3D) networks that are held together by chemical or physical cross-links [1,2]. It possesses the property that can rapidly swell in water without dissolution and is compatible with bio-bodies [3]. Because of these excellent characteristics, hydrogels have attracted considerable attention for applications in the fields of tissue engineering, drug delivery, cell therapy, soft electronics, and soft robotics [2,4,5,6,7,8,9,10]. However, conventional hydrogels limit their applications in passive properties and lack controllability in response to external stimuli [5,7,11,12]. Recently, stimuli-responsive hydrogels whose properties can be adjusted by environmental parameters such as magnetic field (MF), electric field (EF), temperature, pH, and light, have found many potential applications for their enhanced functionalities [13,14,15,16,17].

Magnetic hydrogels, a stimuli-responsive hydrogel, are prepared by incorporating magnetic particles into hydrogels, whose properties can be regulated remotely via an applied MF [18,19]. The magnetic particles embedded in the hydrogels play an important role in the field-responsive behaviors of magnetic hydrogels [11,20,21,22]. Magnetic particles usually contain Fe_3_O_4_, carbonyl iron (CI), and cobalt ferrite (CoFe_2_O_4_). Their type, size, content, and distribution inside the hydrogels (isotropy and anisotropy) all have significant effects on the mechanical characteristics and controllability of magnetic hydrogels [1,22,23,24,25,26,27,28].

Most magnetic hydrogels are made of magnetic nanoparticles (size less than 50 nm), which possess good compatibility and effective interactions with the polymer matrix. The magnetic nanoparticles can deform and displace the gel composite in response to an MF. Bonini et al. prepared a magnetic hydrogel consisting of CoFe_2_O_4_ magnetic nanoparticles and a polyethylene glycol-AAM gel matrix and demonstrated that the nanoparticles were successfully and uniformly distributed in the gel [29]. The presence of nanoparticles has less effect on the excellent hydrophilic properties of the hydrogels; however, they can improve the mechanical strength of the 3D network. No details were reported on how the applied MF deformed the nanomagnetic hydrogel. Konwar et al. developed a simple laboratory technique to synthesize magnetic alginate–Fe_3_O_4_ hydrogel fibers from natural polymer alginate [30]. Both the mechanical and thermal properties of the magnetic alginate–Fe_3_O_4_ hydrogel fibers were improved in comparison with the blank alginate fibers. Hu et al. fabricated strong, tough, and adhesive magnetic hydrogels by embedding a high content of Fe_3_O_4_ (up to 60% with respect to the total weight of the hydrogels) within the PAAM hydrogel [28]. The surface modification of Fe_3_O_4_ was used to functionalize and bond them to the matrix to achieve a relatively high modulus and toughness compared to the pure hydrogel. The high magnetic particle content also helped to achieve a fast field response of the magnetic hydrogel. Lee et al. developed tough, biocompatible, and magneto-responsive nanocomposite hydrogels by the in situ free-radical polymerizations of *N*,*N*-dimethylacrylamide (DMAAM), laponite, and Fe_3_O_4_ [31]. The viscoelastic and mechanical properties of the corresponding hydrogels were investigated with respect to the mixture components. They showed that the optimal magnetic hydrogels have noticeable magnetorheological (MR) behavior, excellent mechanical properties, and good biocompatibility. Pang et al. synthesized magnetic hydrogel scaffolds based on alginate encapsulation of Fe_3_O_4_ [32]. They focused on the dynamic viscoelasticity and MR properties of magnetic hydrogels, aiming at the possible application of tissue engineering scaffolds for their tunable stiffness.

However, the magnetic field-induced stiffness or MR effect of magnetic hydrogels incorporated with nanoparticles is weak because the nanoparticle interactions are dominated by thermal motion. In contrast, micro-sized particles such as CI exhibit stronger MR behavior [22]. Wu et al. prepared isotropic and anisotropic polyvinyl alcohol (PVA) hydrogels containing micron-sized CI through a physically cross-linked cyclic freezing–thawing process [33]. They achieved a significant MR effect of the magnetic hydrogel with up to 230% changes in modulus under an MF of 1.0 T. The embedded CI also enhanced the mechanical properties of PVA hydrogels. Mitsumata et al. developed a magnetic hydrogel consisting of carrageenan and CI [34]. This kind of magnetoelastic gel with 30% magnetic particles exhibited a reversible increase by a factor of 1400 for the storage modulus when subjected to a moderate MF of 0.5 T. Ikeda et al. investigated the effect of magnetic particles on the magnetic responsive properties of carrageenan magnetic hydrogels containing CI, iron oxide, and barium ferrite particles with different diameters. They concluded that the “change in storage modulus exhibited a power dependency against the number of magnetic particles, which was nearly independent of the magnetic particles” [35] (p. 5). Cvek et al. fabricated magnetic hydrogels based on poly (2-ethyl-2-oxazoline) (POX) via living ring-opening cationic polymerization with in situ embedding of the CI [36]. The effect of the CI concentration on the magneto-mechanical activity and biocompatibility of magnetic hydrogels was explored. The hydrogels demonstrated remarkable field-induced stiffening with an increase in the CI concentration in the presence of MF.

Although the mechanical properties of magnetic hydrogels have been widely studied, they are not systematically explored for the effects of the size, content, and distribution structure (isotropy and anisotropy) of the magnetic particles on the magneto-responsive behaviors as well as the swelling properties of the magnetic hydrogels. Bonhome-Espinosa et al. investigated the effect of magnetic nanoparticle concentration on the physical properties of magnetic hydrogels in terms of their macroscopic appearance, microscopic structure, swelling behavior, rheological properties, and gelling time. They found that the mechanical properties of the magnetic hydrogels such as rigidity modulus and the viscoelastic moduli “increased quadratically with nanoparticle content following a square-like function,” while these magnetic hydrogels still have high porousness and swelling capacity [37] (p. 13). Recently, we studied the effect of CI on the rheological and piezoresistive behaviors of isotropic and anisotropic magnetic hydrogels [38]. We found that anisotropic magnetic hydrogels have better performance than isotropic magnetic hydrogels, which exhibit noticeable MR effects, excellent mechanical properties, and high piezoresistive behaviors.

In this study, we aim to explore magnetic hydrogels that possess controllable mechanical and swelling properties. Two magnetic particles of different sizes were selected: Fe_3_O_4_ and CI, which were, respectively, filled into a PAAM hydrogel matrix formed by AAM polymerization and crosslinking. Magnetic hydrogels with magnetic particles of 1, 2, and 3 vol% were prepared. Two types of magnetic hydrogels, isotropy and anisotropy, were designed in this study. The isotropic samples were cured under zero MF, and the anisotropic samples were formed by applying an MF in a direction perpendicular to the thickness of the sample during the curing process. SEM was used to investigate the structure of the magnetic hydrogels. The dynamic viscoelastic properties of all samples were tested and analyzed in strain amplitude sweep mode with strain amplitude changed from 0.01 to 100% at a constant angular frequency of 10 rad/s, and the frequency sweep mode with angular frequency varied from 0.1% to 100 rad/s at a fixed strain amplitude of within the linear strain under different MFs (0 T, 0.5 T) using a commercial MR rheometer. In addition, the swelling properties of all samples were studied. By investigating the effects of the size, content, and distribution inside the hydrogels of magnetic particles on the magneto-responsive and swelling properties of magnetic hydrogels, we are expected to obtain a vast knowledge of the performance of magnetic hydrogels, thus providing a guideline for improving the functionalization of magnetic hydrogels for practical applications.

## 2. Results

### 2.1. Morphology

**Figure 1:**
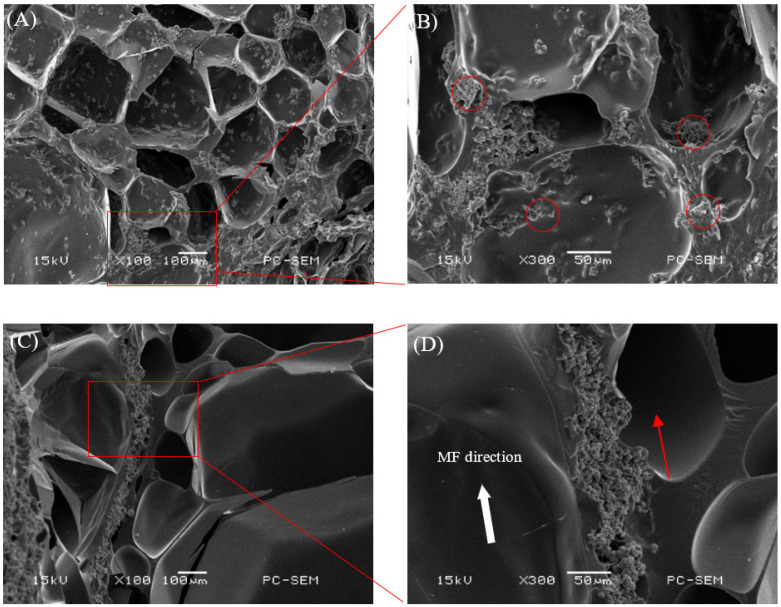
SEM images of cross-section surfaces of the freeze-dried magnetic hydrogels with 2 vol% CI. (**A**) isotropic sample; (**B**) magnification of the red rectangular area in (**A**); (**C**) anisotropic sample; (**D**) magnification of the red rectangular area in (**C**).

**Figure 2:**
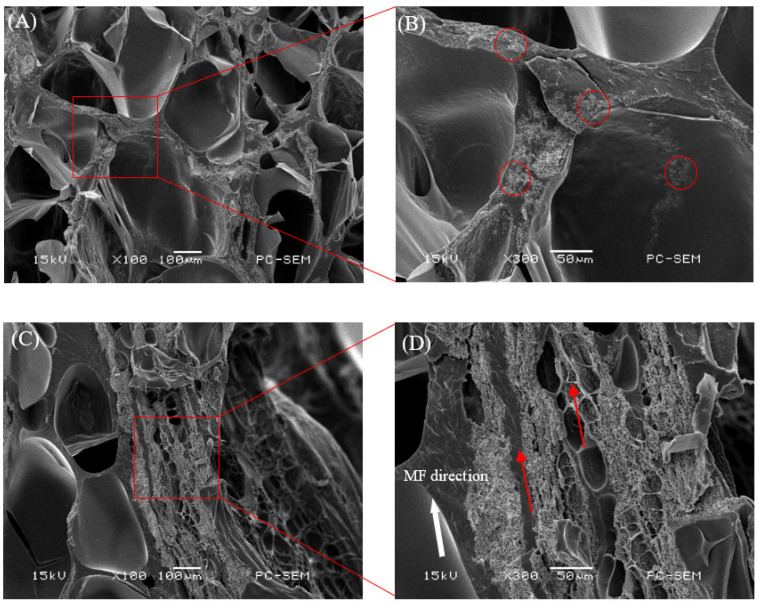
SEM images of cross-section surfaces of the freeze-dried magnetic hydrogels with 2 vol% Fe_3_O_4_. (**A**) isotropic sample; (**B**) magnification of the red rectangular area in (**A**); (**C**) anisotropic sample; (**D**) magnification of the red rectangular area in (**C**).

The isotropic samples were cured under a zero magnetic field, and the anisotropic samples were cured by applying a magnetic field of 0.5 T in the direction perpendicular to the thickness of the samples. The microscopic morphologies of the isotropic and anisotropic samples with 2 vol% CI and Fe_3_O_4_ fillers were examined by SEM, as shown in Figure 1 and Figure 2, respectively. Figure 1A shows the microscopic morphology of the isotropic sample with the CI fillers. The CI particles were randomly distributed within the hydrogel. Figure 1B shows a high-magnification SEM image of the red rectangular area to illustrate that the CI particles were well dispersed in the hydrogel. Figure 1C,D show that the CI fillers form a column/chain-like structure in the anisotropic sample, resulting from the CI particles physically moving their magnetic moments toward the direction of the magnetic field and cured in place to align in a single direction when a magnetic field of 0.5 T is applied. The inner structures of all the samples exhibited an interconnected and three-dimensional porous network architecture, indicating good miscibility between the magnetic particles and the hydrogel. For these isotropic samples with Fe_3_O_4_ fillers, their particle distribution is similar to that of CI particles, which are randomly dispersed inside the matrix gels, except that Fe_3_O_4_ particles tend to agglomerate into a small group (Figure 2A,B). However, the magnetic hydrogels with Fe_3_O_4_ cured under magnetic fields showed different forms of particle structure, where the Fe_3_O_4_ fillers non-uniformly accumulated together like porous columns along the direction of the magnetic field (Figure 2C,D). There are no apparent chain structures in anisotropic magnetic hydrogels with Fe_3_O_4_, probably because Fe_3_O_4_ is a nanoparticle, while CI is a micro-particle.

### 2.2. The Rheological Properties of Magnetic Hydrogels with Fe_3_O_4_

The rheological properties of magnetic hydrogels with 1, 2, and 3 vol% of Fe_3_O_4_ with isotropic and anisotropic structures were characterized by measuring the dynamic storage modulus (G′) and loss modulus (G′) subject to different MFs. The loss factor (tan δ), which is the ratio of G″ to G′, represents the ratio of dissipated energy stored to retained energy during the deformation of materials and shows the mechanical damping of the viscoelastic material. The tan δ reflects the difference in strength between the loss and storage modules.

#### 2.2.1. Strain Amplitude Sweep

**Figure 3:**
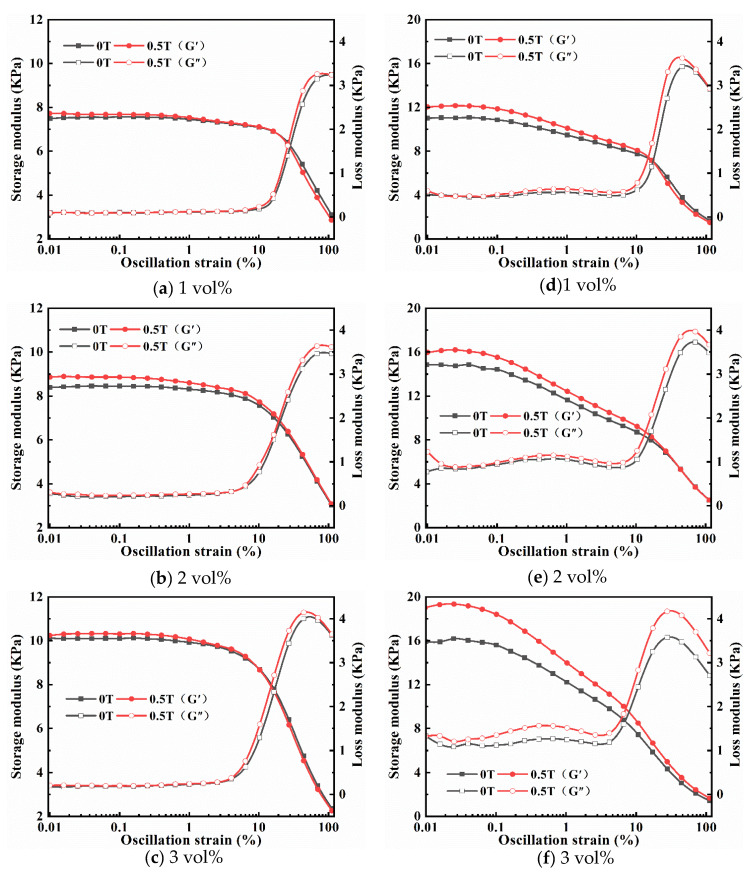
Strain amplitude dependence of the G′ and G″ of isotropy (**a**–**c**) and anisotropy (**d**–**f**) samples with Fe_3_O_4_ of 1 vol%, 2 vol% and 3 vol% under 0 T and 0.5 T at a fixed frequency of 10 rad/s.

In the strain amplitude sweep mode, the G′ and G″ of the magnetic hydrogels were tested under varying strains ranging from 0.01% to 100% at a constant frequency of 10 rad/s with and without an applied MF. Figure 3 shows the strain amplitude dependence of the G′ and G″ of isotropic (a–c) and anisotropic (d–f) magnetic hydrogels with different Fe_3_O_4_ contents in the absence (0 T) and presence (0.5 T) of MF. Overall, the G′ values are higher than those of G″ for all samples, which is typical behavior of hydrogels. As the strain amplitude increases, G ‘remains nearly fixed at low strain amplitude levels, indicating a well-established network structure of the hydrogels, but G′ sharply decreases when the strain amplitude exceeds a critical value. However, G″ remained almost constant at lower strain amplitudes, and then increased up to a maximum (peak value), followed by a sharp decrease. The linear viscoelastic (LVE) region occurs where the dynamic modulus is independent of the strain, while outside the region is the nonlinear viscoelastic (NLVE) region, where the internal structure of the magnetic hydrogels suffers from irreversible deformation and breakage. It can be observed that with the increase in the content of Fe_3_O_4_, the LVE ranges of isotropic and anisotropic magnetic hydrogels all decrease. Comparing Figure 3a–c with Figure 3d–f, the LVE range of anisotropic magnetic hydrogels is lower than that of the isotropic for the same filler content in the absence (0 T) and presence (0.5 T) because the Fe_3_O_4_ in anisotropic magnetic hydrogels tends to accumulate column-like structures along the direction of the MF in the curing process (as shown in Figure 2C,D). However, MF had little effect on the LVE zone of all the samples. Finally, a strain of 0.1% was selected for the angular frequency sweep measurement considering the linear viscoelastic region for isotropic and anisotropic magnetic hydrogels.

#### 2.2.2. Frequency Sweep

**Figure 4:**
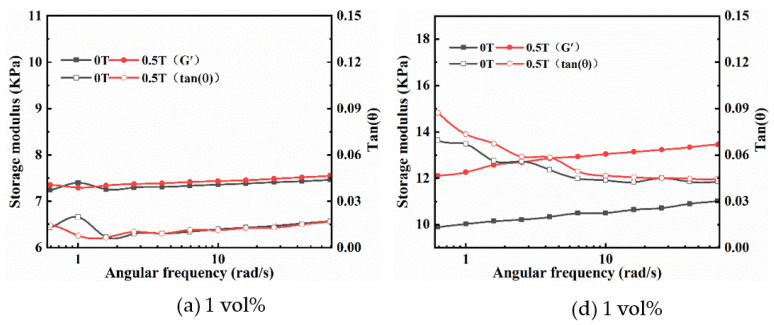

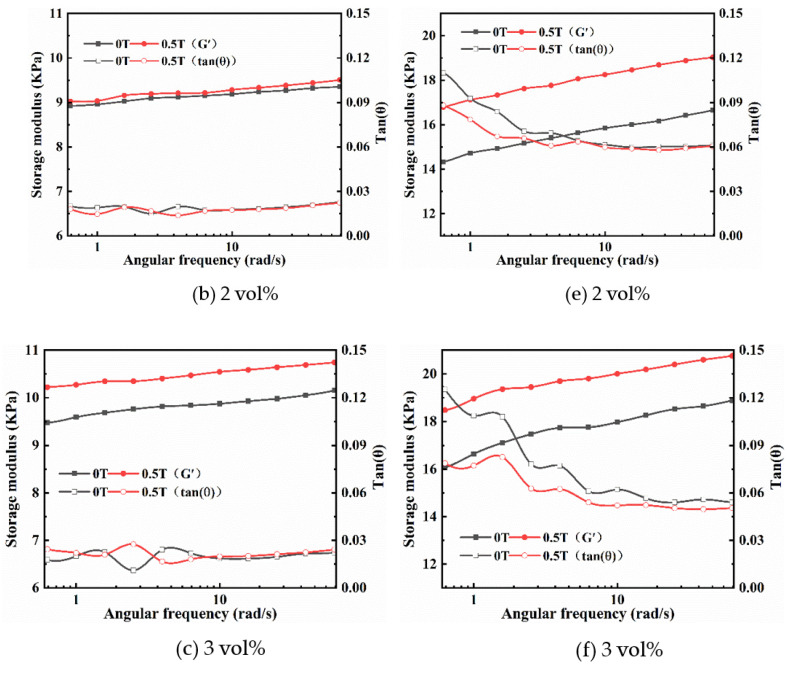
Angular frequency dependence of the G′ and tan δ of isotropy (**a**–**c**) and anisotropy (**d**–**f**) samples with Fe_3_O_4_ of 1 vol%, 2 vol% and 3 vol% under 0 T and 0.5 T at a fixed strain amplitude of 0.1%.

In the frequency sweep mode, the storage modulus (G′) and loss factor (tan δ = G″/G′) of magnetic hydrogels were tested under varying frequencies ranging from 0.1 rad/s to 100 rad/s at a constant strain amplitude of 0.1% within the LVE region. Figure 4 displays the relationships between the angular frequency and the G′ and tan δ of isotropic (a–c) and anisotropic (d–f) magnetic hydrogels with different Fe_3_O_4_ contents under different MFs (0 T, 0.5 T) at a fixed strain amplitude. Overall, a similar change trend in G′ was observed for all samples, where G′ increased gradually with increasing frequency; however, the loss factor tan δ remained nearly constant for isotropic samples and the loss factor for anisotropic samples initially decreased and then gradually maintained a lower level. Figure 4a–c show that the tan δ of the isotropic sample under 0.5 T is almost the same as those under 0 T, which suggests that the tan δ of the isotropic sample is not affected by the magnetic field. However, the tan δ of the anisotropic sample of 2 vol% and 3 vol% Fe_3_O_4_ under 0.5 T is larger than these under 0 T and for the anisotropic sample of 1 vol% Fe_3_O_4_ under 0.5 T, tan δ is slightly less than these under 0 T, as shown in Figure 4d–f. This indicates that the loss factor of the anisotropic sample is affected by the magnetic field, and this influence is greater with an increase in the Fe_3_O_4_ content. The loss factor for all sample values was lower than 1 (tan δ < 1), which suggested that the elastic behavior (solid-like) was more prevalent than the viscous (liquid-like) nature of the magnetic hydrogels. Moreover, the tan δ for anisotropic samples is higher for isotropic samples because anisotropic samples form column-like structures along the direction of the MF in the curing process.

#### 2.2.3. MR Effect

**Figure 5:**
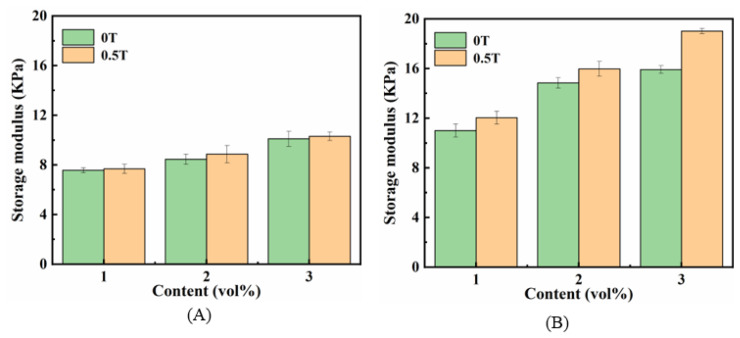
Effects of the contents of Fe_3_O_4_ on the G′ of isotropy (**A**) and anisotropy (**B**) samples within LVE region (f = 10 rad/s, B = 0 T and 0.5 T).

Generally, the MF-induced changes in the mechanical properties of magnetic hydrogels can be presented as the MR effect, which is calculated based on G′ within the LVE range at an applied MF and non-MF. The specific expression of the MR effect is as follows [39]:

$$\mathrm{MR}\;\mathrm{effect}\;(\%)=\frac{G_{\mathrm{MF}}^{\prime }-G_{0}^{\prime }}{G_{0}^{\prime }}\times 100\%$$

where $G_{\mathrm{MF}}^{\prime }$ is the storage modulus in the presence of an MF, $G_{0}^{\prime }$ is the storage modulus without applied MF, and $G_{\mathrm{MF}}^{\prime }-G_{0}^{\prime }$ is the magneto-induced storage modulus. Typically, the MR effect is a function of the MF and particle concentrations. Figure 5 shows the effect of content Fe_3_O_4_ on the G′ of isotropic (A) and anisotropic (B) magnetic hydrogels with and without an applied MF at a frequency of 10 rad/s within the LVE region. The results reveal that the G′ of all samples increases with increasing Fe_3_O_4_ concentration, and the applied MF of 0.5 T has a slight enhancement on the G′ values of all samples. Comparing the results of the anisotropic magnetic hydrogels (Figure 5B) with those of isotropic magnetic hydrogels (Figure 5A), it can be seen that the G′ values of anisotropic samples are apparently higher than that of the isotropic structure, and the MR effect of the anisotropic magnetic hydrogel can reach up to 19.51% with a content of 3 vol%. It is apparent that the magnetic hydrogels cured under an MF possessing anisotropic structures would have better magneto-responsive properties than those of isotropic magnetic hydrogels without curing. However, for magnetic hydrogels with Fe_3_O_4_ fillers, the MR effect is still weak.

### 2.3. The Rheological Properties of Magnetic Hydrogels with CI

Following the procedure described in Section 3.2, a similar rheological testing protocol was performed on the magnetic hydrogels filled with CI micro-sized particles. The effects of the CI concentration and formed structures on the rheological properties of the magnetic hydrogels were investigated. The results were compared with those of the magnetic hydrogel with Fe_3_O_4_.

#### 2.3.1. Strain Amplitude Sweep

**Figure 6:**
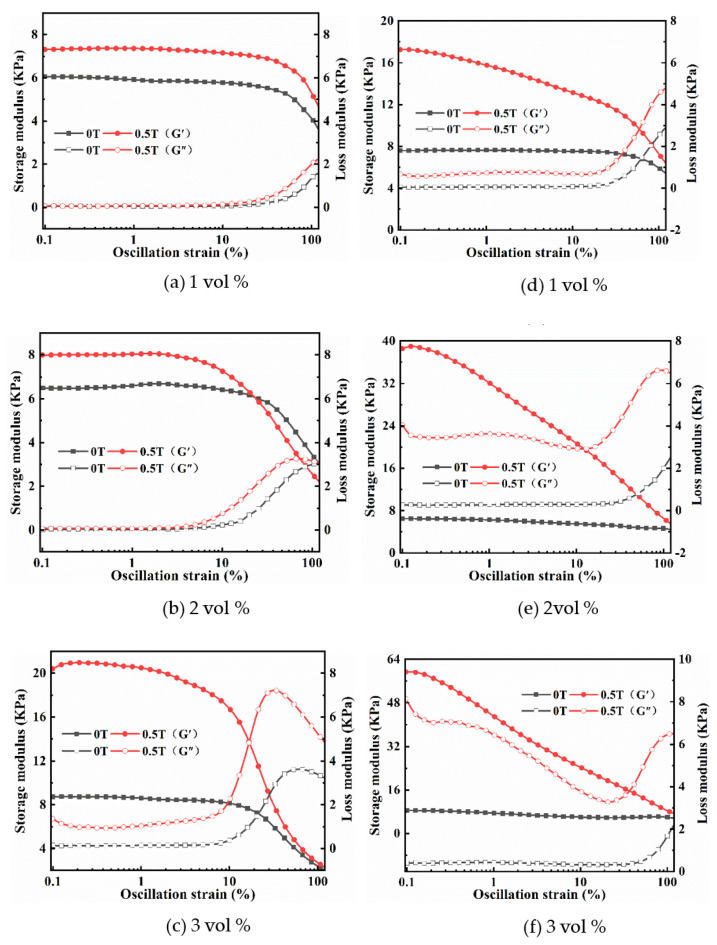
Strain amplitude dependence of the G′ and G″ of isotropy (**a**–**c**) and anisotropy (**d**–**f**) samples with CI of 1 vol%, 2 vol% and 3 vol% under 0 T and 0.5 T at a fixed frequency of 10 rad/s.

Figure 6 shows strain amplitude dependence of the G′ and G″ of with different CI content under the absence (0 T) and presence (0.5 T) of MF. Overall, the G′ values are higher than those of G″ and the changing trends of G′ and G″ with strain amplitude increasing for all samples with CI, which are similar to those with Fe_3_O_4_. Comparing the results in Figure 6a–f, it can be seen that the LVE ranges for both isotropic and anisotropic magnetic hydrogels decrease with the concentrations of CI. The LVE ranges of anisotropic magnetic hydrogels are lower than that of the isotropic for the same filler content under the absence and presence, which is due to the CI in anisotropic magnetic hydrogels forming a chain-like structure in the curing process. However, the MF has apparent effects on the LVE zone of magnetic hydrogels filled with CI in contrast to those with Fe_3_O_4_.

#### 2.3.2. Frequency Sweep

**Figure 7:**
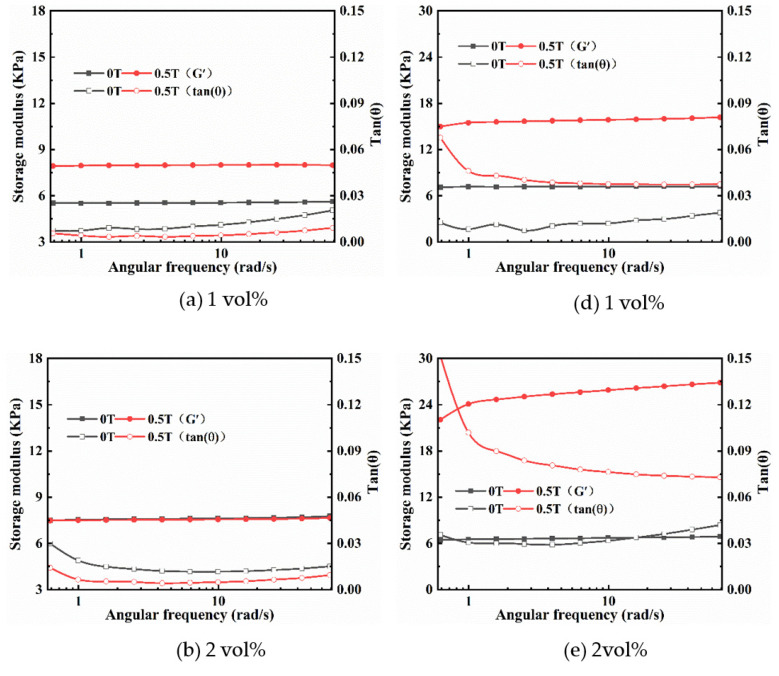

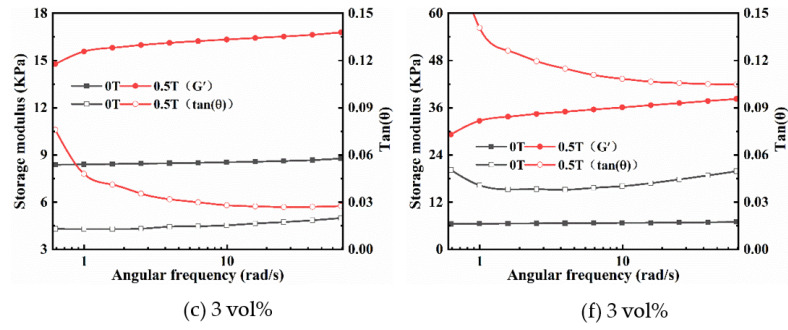
Angular frequency dependence of the G′ and tan δ of isotropy (**a**–**c**) and anisotropy (**d**–**f**) samples with CI of 1 vol%, 2 vol% and 3 vol% under 0 T and 0.5 T at a fixed strain amplitude of 0.1%.

A strain of 0.1% was selected for the angular frequency sweep measurement considering the LVE region for the isotropic and anisotropic magnetic hydrogels. In the frequency sweep mode, the G′ and tan δ of magnetic hydrogels were tested under varying frequencies ranging from 0.1 rad/s to 100 rad/s at a constant strain amplitude of 0.1% subject to two different MFs (0 T and 0.5 T). The results for G′ and tan δ as a function of angular frequency are plotted in Figure 7 for isotropic (a–c) and anisotropic (d–f) magnetic hydrogels with different CI contents under different MFs. It can be found that the G′ of all sample fields are almost independent of frequencies under zero MF, but a slight increase with frequencies when an MF of 0.5 T was applied. Additionally, the applied MF also increased G′ for all samples. Moreover, the changes in G′ significantly increased with the content of CI. Compared with isotropic magnetic hydrogels, the effects of MF on the viscoelastic properties of anisotropic magnetic hydrogels are more profound. In the presence of an external MF, the storage modulus and loss factors of anisotropic samples are obviously higher than those of isotropic samples.

#### 2.3.3. MR Effect

**Figure 8:**
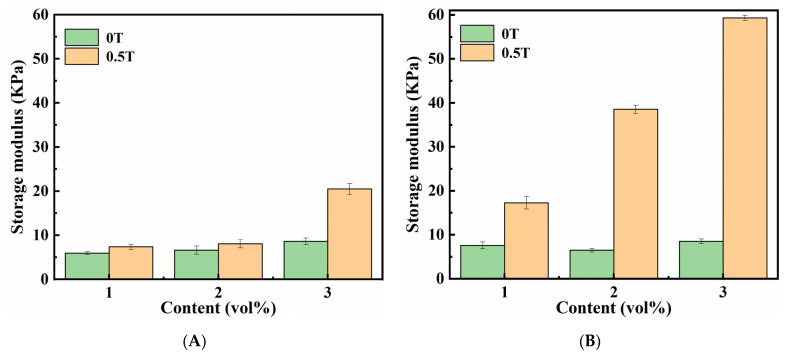
Effects of the contents of CI on the G′ of isotropy (**A**) and anisotropy (**B**) samples within LVE region (F = 10 rad/s, B = 0 T and 0.5 T).

To demonstrate the MR effect of magnetic hydrogels with CI, the values of G′ for the isotropic (A) and anisotropic (B) magnetic hydrogels with and without an applied MF at a frequency of 10 rad/s and a strain amplitude of 1% are presented in terms of the CI concentrations in Figure 8. The results revealed that: (1) there is a slight change in G′ with the change in particle concentrations without MF; however, a significant change with an applied MF, (2) the G′ of all samples was enhanced when an MF was applied, and (3) the G′ of anisotropic magnetic hydrogels was significantly higher than that of isotropic magnetic hydrogels. The MR effect of the anisotropic magnetic hydrogel with 3 vol% CI can reach up to 597.62% at an applied MF strength of 0.5 T, while the isotropic magnetic hydrogel can only achieve 138.05% under the same conditions. This can be attributed to the CI in anisotropic magnetic hydrogels forming a chain-like structure during the curing process. When these anisotropic magnetic hydrogels are subjected to an applied MF, more strength is required to deform the materials perpendicular to the direction of the field. All the results demonstrated that the storage modulus G′ can be enhanced by increasing the filler content, forming a chain-like structure, and applying an external MF. The storage modulus G′ of the magnetic hydrogels can be controlled by the MF, exhibiting their potential application as variable stiffness devices.

By comparing the effects of Fe_3_O_4_ and CI on the rheological properties of magnetic hydrogels, we found that the influences of the content and structures (isotropic and anisotropic) of fillers on the magneto-responsive properties of magnetic hydrogels have similar change trends. The significant difference is the MR effect, which is referred to as the relative change in the storage modulus in comparison with the initial modulus of magnetic hydrogels within the LVE region. Magnetic hydrogels filled with CI have a much higher MR effect than magnetic hydrogels filled with Fe_3_O_4_. The MR effects were 2.07% and 19.51% for the isotropic and anisotropic magnetic hydrogels with Fe_3_O_4_, respectively, while 138.05% and 597.62% for those with a CI content of 3 vol% at an applied MF strength of 0.5 T. This difference is due to the size difference between the Fe_3_O_4_ and CI fillers.

### 2.4. Swelling Behavior

The swelling behavior of hydrogels is that their volume is increased by absorbing a large amount of water without dissolution because of the structure of hydrophilic polymer chains with 3D networks. In the swelling process, on the one hand, the water solvent tries to penetrate into the polymer to expand its volume. On the other hand, the volume expansion of the cross-linked polymer causes the network molecular chains to extend to the three-dimensional space. Then, the molecular network structure is forced to contract because it is stressed and produces elastic contraction energy. When these two opposite tendencies are against each other, an equilibrium of swelling is reached. The swelling ratio in the swelling process is defined as the dynamic swelling ratio (DSR). When swelling is in equilibrium, the swelling ratio is defined as the equilibrium swelling ratio (ESR). The influence of the content, size, and distribution inside the hydrogels of magnetic particles on the swelling behavior of the hydrogels was analyzed.

#### 2.4.1. Swelling Behavior of Magnetic Hydrogels with Fe_3_O_4_

**Figure 9:**
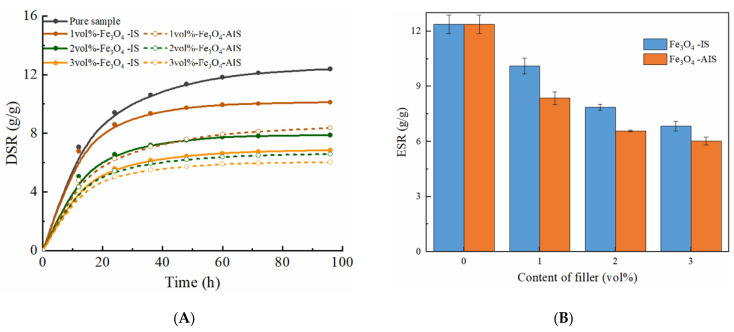
(**A**) The DSR plotted as a function of the swelling time for dried samples with different Fe_3_O_4_ content in deionized water. (**B**) The effects of Fe_3_O_4_ content on ESR. IS: isotropic samples, AIS: anisotropic samples.

Figure 9A shows the swelling behavior of the samples with different Fe_3_O_4_ contents compared to the pure reference samples. It is evident that DSR is a function of the swelling time. All the samples began to swell rapidly, then gradually flattened out, and finally reached the swelling equilibrium, which is the typical swelling behavior of hydrogels. We also observed that the DSR for all magnetic samples was lower than that of pure samples. On the other hand, we found that the ESR showed a systematically decreasing tendency as the amount of Fe_3_O_4_ in the matrix was further increased, as shown in Figure 9B. The above results can be attributed to an attractive interaction between Fe_3_O_4_ and the PAAM polymer matrix, potentially involving the -NH_2_ hydrophilic group on the surface of the PAAM chain. The interaction of Fe_3_O_4_ and -NH_2_ led to a decrease in the interaction between -NH_2_ and water molecules. Thus, the ability to form hydrogen bonds between the PAAM chain and water molecules decreases, which makes it difficult for water molecules to enter the hydrogel network. At the same time, the interaction between Fe_3_O_4_ and the PAAM chain causes a tighter network structure. This makes the hydrogel network have a stronger elastic contraction and is more difficult to expand in volume. Thus, the capacity for water absorption becomes weaker, and the swelling capacity is reduced. Hence, the higher the content of Fe_3_O_4_ and the greater the interaction, leading to an effective decrease in the swelling ability. These observations are consistent with those of other researchers [40]. In summary, these water swelling experiments showed that the introduction of nanoparticles Fe_3_O_4_ into the PAAM matrix affects its swelling behavior. Therefore, we conclude that the swelling ability of magnetic hydrogels can be tuned by varying the amount of Fe_3_O_4_.

#### 2.4.2. Swelling Behavior of Magnetic Hydrogels with CI

**Figure 10:**
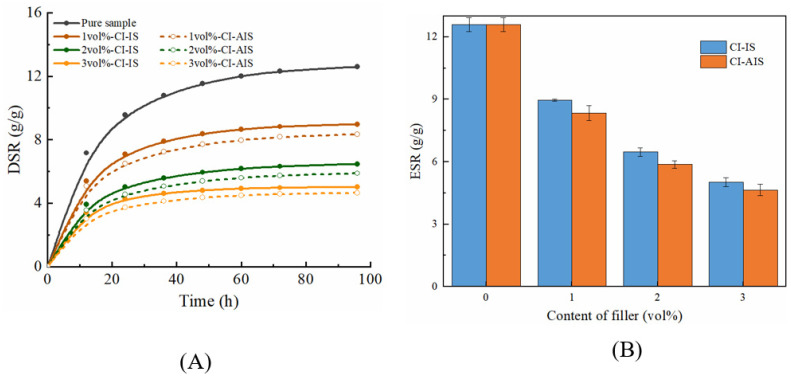
(**A**) The DSR plotted as a function of the swelling time for dried samples with different CI content in deionized water. (**B**) The effects of CI content on ESR.

The swelling behavior of magnetic hydrogels filled with CI was also explored. The effects of time on the DSR of these samples with different CI contents are shown in Figure 10A. The effects of the CI content on the ESR are shown in Figure 10B. Overall, for the samples with CI, the DSR increases quickly during the initial period, and then gradually flattens out as time increases, finally reaching the swelling equilibrium, which is similar to that of Fe_3_O_4_. The ESR also gradually decreases with an increase in the CI content. These results were caused by the fact that the hydrophilic structure of the PAAM chain is affected by CI, which is similar to the samples with Fe_3_O_4_.

However, comparing the swelling ability of the samples with Fe_3_O_4_, we found that the swelling ability of the samples with CI was weaker than that of the samples with Fe_3_O_4_. This may be because the size of CI is larger than that of Fe_3_O_4_, and the number of CIs in the matrix is less than the number of Fe_3_O_4_ particles at the same volume content. As a result, fewer physical cross-linking points between the particles and matrix decreased the cross-linking density and reduced the swelling capacity. By comparing the swelling ability of isotropic and anisotropic samples, we found that the swelling ability of isotropic samples was greater than that of anisotropic samples. This may be because the particles in the isotropic samples were uniformly dispersed, while the particles in the anisotropic samples were clustered, which led to more cross-linking points between the particles and the matrix in the isotropic sample. Thus, the isotropic samples exhibit a stronger swelling capacity. The swelling ability of magnetic hydrogels can be controlled by varying the amount, size, and distribution of the fillers. On the other hand, it retains a considerable swelling function even with high fillers.

## 3. Materials and Methods

### 3.1. Material Preparations

#### 3.1.1. Raw Materials

Table 1 lists the raw materials and their producers for preparing the magnetic hydrogels. AAM, *N*-methylenebisacrylamide (MBAA), ammonium persulfate (APS), and *N*,*N*,*N*′,*N*′-tetramethylethylenediamine (TEMED) were used as monomers, crosslinking agents, initiators, and accelerators, respectively, to prepare the hydrogel matrix. CI (average diameter 1–3 μm) and Fe_3_O_4_ (average diameter 1–10 nm) were selected as fillers for the magnetic hydrogels. Methacrylic acid (MAA, 99%), (VTMOS 98%), and ethanol (98.8%) were purchased for surface pretreatment of CI.

#### 3.1.2. Surface Pre-Treatment of CI

We pre-treated the surface of CI by coating with silica to prevent their oxidation before preparing the magnetic hydrogels [40]. First, the CI, ethanol solution, and MAA were added to a beaker and then treated by the ultrasonic dispersion method for 30 min at 25 °C. Next, VTMOS was poured into the mixture with full mechanical stirring at 400 rpm for 20 min. Finally, we obtained the silica-coated CI washed with ethanol until the supernatant was clear and dried for 48 h in a vacuum drying oven.

#### 3.1.3. Preparation of Magnetic Hydrogels

**Figure 11:**
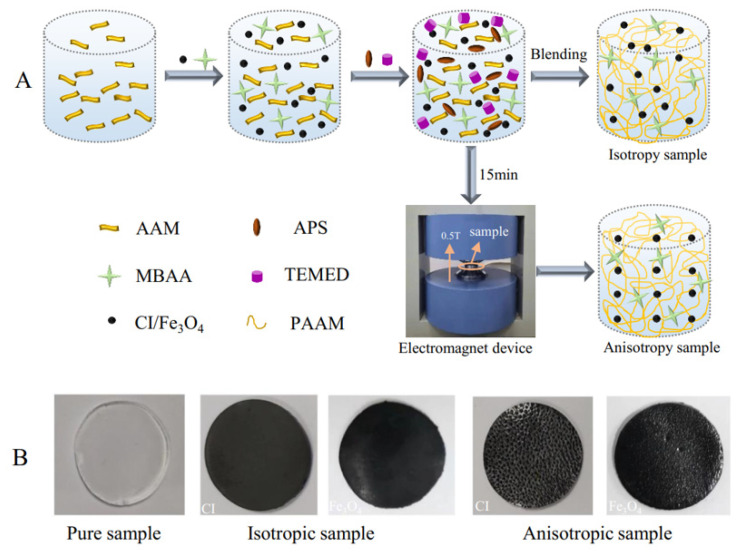
(**A**) Schematic illustration of the preparation procedure of magnetic hydrogels. (**B**) Pictures of pure hydrogel and magnetic hydrogels with isotropy and anisotropy of 2 vol% CI/Fe_3_O_4_.

Magnetic hydrogels were synthesized by free-radical polymerization of AAM and fillers in aqueous solution. The main process for obtaining the magnetic hydrogels is shown in Figure 11A. Briefly, the AAM monomer was dissolved in deionized water and added together with magnetic particles of 1, 2, and 3 vol%. MBAA as the crosslinking agent, APS as the initiator, and TEMED as the accelerator were then added sequentially to the mixture solution with continuous stirring. The above mixture was poured into two aluminum molds: one was evenly blended without an MF for 15 min to obtain isotropic samples, and the other was cured under 0.5 T for 15 min to form anisotropic samples. Figure 11B shows macro-scale photographs of the pure sample and isotropic and anisotropic samples with 2 vol% of CI/Fe_3_O_4_. The matrices for preparing the material samples with various components are listed in Table 2.

### 3.2. Characterizations

#### 3.2.1. Morphology

SEM (JSM-6510F, JEOL, Tokyo, Japan) was used to observe the microstructural morphology of the fillers in the magnetic hydrogels. The hydrogel specimens frozen with liquid nitrogen were fractured to expose the cross-section and observe their internal structure. For the anisotropic samples, the brittle fractures were along the alignment direction of the particle chains.

#### 3.2.2. Rheological Properties

**Figure 12:**
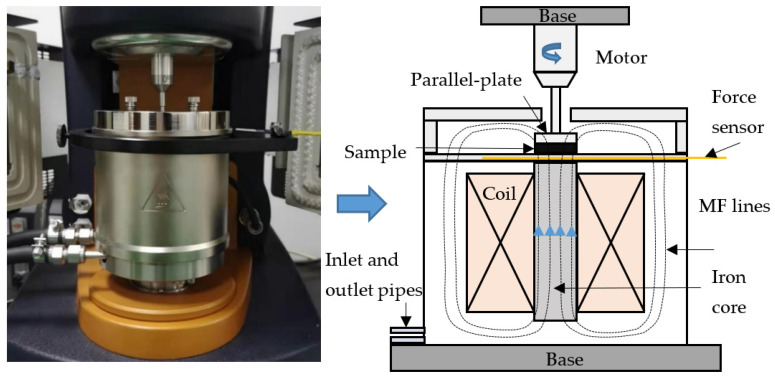
The photo and schematic of MR properties testing system.

The dynamic viscoelastic properties of all samples were tested using a rotational rheometer (DHR-2, TA Instrument, New Castle, DE, USA) with a 20 mm diameter parallel-plate system and a controlled magnetic cell. The magnetic cell can generate a uniform magnetic flux density in the 1.0 mm gap from 0 to 1.0 T by adjusting the coil current from 0 to 2 A. The direction of the MF was perpendicular to the shear direction during the dynamic tests. A photograph and a schematic of the rheological properties testing system are shown in Figure 12. In this study, the distance between two parallel plates (testing gap).was maintained at 800 µm throughout the experiment, and the thickness of the tested circular samples with a diameter of 20 mm was made into 1.0 mm. First, the strain amplitude sweep mode was carried out to investigate the nonlinear viscoelastic (NLVE) properties of magnetic gels with strain amplitude changed from 0.01 to 100% at a constant angular frequency of 10 rad/s. The frequency sweep mode with angular frequency varied from 0.1 to 100 rad/s at a fixed strain amplitude within the linear strain range was performed to characterize the dynamic behaviors of the magnetic gels as a function of frequency. Both sweep tests of the strain amplitude and frequency were carried out under two magnetic flux densities of 0.0 T and 0.5 T. All the measurements were conducted at a room temperature of 25 °C.

#### 3.2.3. Swelling Studies

The swelling behavior was studied by immersing freeze-dried samples in deionized water at 25 °C. The samples were removed from the water and weighed at 12 h intervals until fully swollen. The samples were freeze-dried using a freeze-dryer (LEJ-12D, China). The equilibrium swelling ratio (ESR) of the magnetic hydrogels was obtained from the average of three measurements according to the formula [41]:

$$ESR=\frac{W_{s}-W_{d}}{W_{d}}$$

where $W_{s}$ and $W_{d}$ are the weights of the samples fully swollen in aqueous solutions and in the dry state, respectively.

The dynamic swelling ratio (DSR) of magnetic hydrogels was expressed:

$$DSR=\frac{W_{t}-W_{d}}{W_{d}}$$

where $W_{t}$ are the weight of the samples at time *t*.

## 4. Conclusions

In summary, we presented a comprehensive study on the effects of the size, content, and structure (isotropic and anisotropic) of magnetic particles on the magneto-responsive and swelling properties of magnetic hydrogels. Two types of magnetic hydrogels incorporating Fe_3_O_4_ and CI were successfully synthesized by the free-radical polymerization of AAM. The magneto-responsive behaviors of the magnetic hydrogels in terms of particles of 1 vol%, 2 vol%, and 3 vol% under MF of 0 T and 0.5 T were investigated. SEM images showed that Fe_3_O_4_ and CI formed an anisotropic structure in the hydrogel when they were cured under an MF. The CI tends to form chain-like structures, whereas Fe_3_O_4_ is likely to accumulate along the applied MF direction. The MR effect of the anisotropic samples is apparently higher than that of the isotropic samples, and the samples with CI exhibit noticeable MR effects than those with Fe_3_O_4_. These results show that G′ can be enhanced by increasing the filler content and size, forming an anisotropic structure by applying an external MF. Magnetic hydrogels fabricated using CI possess a high potential for use in variable-stiffness devices. In addition, the swelling study of the hydrogels filled with Fe_3_O_4_ or CI demonstrated that magnetic hydrogels not only have field response properties but also the swelling ability can be tuned by varying the amount and size of the fillers and still retain considerable swelling function even with high fillers.

## Figures and Tables

**Table 1:** The raw materials and its producer for preparing magnetic hydrogels.

Functions	Raw Materials	Producers
Hydrogel matrix	AAM	Sinopharm Chemical Reagent Co., Ltd., Shanghai, China
	MBAA	Sinopharm Chemical Reagent Co., Ltd., Shanghai, China
	APS	Sinopharm Chemical Reagent Co., Ltd., Shanghai, China
	TEMED	Sinopharm Chemical Reagent Co., Ltd., Shanghai, China
Magnetic particles	CI with an average diameter ranges from 1 to 3 μm	Jiangsu Tianyi Ultra-fine metal powder Co., Ltd., Huaiyin, China
	Fe_3_O_4_ with an average diameter ranges from 1 to 10 nm	Sinopharm Chemical Reagent Co., Ltd., Shanghai, China
Surface pre-treatment of CI	MAA (99%)	Sigma-Aldrich, St. Louis, MO, USA
	VTMOS (98.0%)	Sigma-Aldrich, St. Louis, MO, USA
	Ethanol (99.8%)	Sinopharm Chemical Reagent Co., Ltd., Shanghai, China

**Table 2:** Material components.

S. No.	CI (vol%)	Fe_3_O_4_ (vol%)	CI (g)	Fe_3_O_4_ (g)	AAM (mL)	MBAA (mL)	APS (mL)	TEMED (µL)
Isotropic samples								
1	1	0	1.78	0	20	1.6	0.8	20
2	2	0	3.60	0	20	1.6	0.8	20
3	3	0	5.45	0	20	1.6	0.8	20
4	0	1	0	1.17	20	1.6	0.8	20
5	0	2	0	2.37	20	1.6	0.8	20
6	0	3	0	3.59	20	1.6	0.8	20
Anisotropic samples								
7	1	0	1.78	0	20	1.6	0.8	20
8	2	0	3.60	0	20	1.6	0.8	20
9	3	0	5.45	0	20	1.6	0.8	20
10	0	1	0	1.17	20	1.6	0.8	20
11	0	2	0	2.37	20	1.6	0.8	20
12	0	3	0	3.59	20	1.6	0.8	20

## Data Availability

Data available upon request.
